# Supplementary material for: Education-related inequality in overweight and obesity among Mongolian adults
Source: PLOS Glob Public Health. 2025 Jun 17;5(6):e0004723. doi: 10.1371/journal.pgph.0004723 (PMC12173372; doi:10.1371/journal.pgph.0004723)
Supplement: Attachment — Submitted filename: RevComs.docx [file pgph.0004723.s001.docx]

This paper is well-executed and places itself effectively within the related literature. The methodology is sound, and the analysis is well conducted. However, the paper’s contribution to the field is not immediately clear, as it does not sufficiently emphasize how it advances current knowledge or offers new insights. To enhance its impact, the authors should focus on clearly articulating the study’s unique value and ensuring it is prominently highlighted in the discussion. Several areas could benefit from revision and clarification:

1. **Rank Variable for SES:** While using years of schooling to measure socioeconomic status can provide valuable insights, it is typically a fallback when income data is unavailable. Income is generally a more robust and sensitive rank variable due to its greater variability across populations and its consistent relationship with obesity. The statement on p.7, line 164, referring to education as “stable over time,” needs further justification. Stability is not always an advantage in this context, as income's dynamic nature reflects current socioeconomic conditions more accurately. Results using income as the rank variable should be presented and compared, and the preference for education over income should be better justified.
2. **Definition of "Location":** The term "location" on p.7, line 170, seems to imply detailed geographic information, but the study only differentiates between rural and urban areas. This distinction should be clarified to avoid misinterpretation.
3. **Alcohol Consumption Variable:** The variable indicating alcohol consumption (p.7, line 177) appears to consider whether a person consumed alcohol more than once in the past year. This threshold is unlikely to meaningfully impact SES inequality in obesity. Alternative measures, such as binge drinking or more frequent recent alcohol use, would be more appropriate.
4. **Decomposition Analysis:** The decomposition results indicate contributions greater than 100%. This needs to be explained thoroughly, including how these results are calculated. In general, tables should include all key components (e.g., concentration index, elasticities, and factor contributions) for transparency and completeness.
5. The sentence on p.4, lines 87–89, is unclear and needs to be rewritten for better comprehension.
6. **Paper Structure**: Include a paragraph at the end of the introduction section outlining the structure of the paper, guiding readers through its organization.
7. **Discussion Section:** A significant portion of the discussion section would be better suited for the introduction and literature review, where it could help frame the study’s research question and relevance.
8. **Conclusion:** The conclusion is too brief and does not adequately summarize the study’s findings. It should discuss the key insights, such as why obesity is more prevalent among women in lower SES groups while the reverse is true for men, and explicitly state what questions the study addresses.
9. **Policy Recommendations:** The policy recommendations are underdeveloped and should provide more detailed, actionable suggestions based on the study’s findings.

If the authors address the points raised, the paper would potentially be considered for publication. I therefore recommend a **REVISE AND RESUBMIT**.
