# Supplementary material for: Education-related inequality in overweight and obesity among Mongolian adults
Source: PLOS Glob Public Health. 2025 Jun 17;5(6):e0004723. doi: 10.1371/journal.pgph.0004723 (PMC12173372; doi:10.1371/journal.pgph.0004723)
Supplement: Attachment — Submitted filename: Response letter to reviewers.docx [file pgph.0004723.s003.docx]

PGPH-D-24-02309

Dear Academic Editor and Reviewers,

Thank you for giving us the opportunity to submit a revised draft of our manuscript titled “Socioeconomic inequality in overweight and obese Mongolian adults: a decomposition approach” to the journal “*PLOS Global Public Health”.* We appreciate the time and effort that you and the reviewers have made to provide valuable feedback on our manuscript. We are grateful to the reviewers for their insightful comments on our manuscript. We have been able to incorporate changes to reflect most of the comments provided by the reviewers. We have made the track changes within the manuscript.

Sincerely,

Authors

**Editor’s comment**

- **Comment 1:** In the online submission form, you indicated that "The data underlying the results presented in the study are available from the National Center for Communicable Diseases of Mongolia."

All PLOS journals now require all data underlying the findings described in their manuscript to be freely available to other researchers, either 
1. In a public repository, 
2. Within the manuscript itself, or 
3. Uploaded as supplementary information.

***Response 1:*** *Thank you for drawing our attention to the data availability statement in the online submission form. Upon review, we noticed that our initial wording contained an error, as it inaccurately referred to the dataset using the phrase "underlying finding." We apologize for this oversight and appreciate the opportunity to correct it. We believe updated wording accurately reflects the data's accessibility and removes any ambiguity.*

*We will rewrite this sentence as follows in the online form “The dataset used for this study cannot be made publicly available because the data is restricted by the National Center for Communicable Disease (NCCD) of Mongolia, the owner of the Mongolian National Tuberculosis Prevalence Survey (MNTPS) (dataset available at* [*https://tubis.nccd.gov.mn*](https://tubis.nccd.gov.mn)*); however, it is available from the NCCD upon a reasonable request. Requests should be submitted to the scientific review committee at the National Center for Communicable Disease, Ulaanbaatar, Mongolia (Email:* [*scientificcommittee@nccd.gov.mn*](mailto:scientificcommittee@nccd.gov.mn)*).”*

**Comment 2:** Please provide separate figure files in .tif or .eps format.

***Response 2:*** *The figure files have been updated.*

- **Comment 3:** Please provide an Author Summary. This should appear in your manuscript between the Abstract (if applicable) and the Introduction, and should be 150–200 words long. The aim should be to make your findings accessible to a wide audience that includes both scientists and non-scientists.

***Response 3:*** *Thank you for your suggestion. We wrote the author summary as follows:*

*Author summary*

*Line 58-74, p-3*

*Obesity is a growing health challenge worldwide and contributes to serious diseases like heart disease, diabetes, and cancer. In Mongolia, where the population is rapidly transitioning from a traditional lifestyle to a more urban and modern way of living, obesity rates are increasing. However, little is known about how obesity affects people from different social and economic backgrounds in Mongolia. In this study, we analyzed data from a National Tuberculosis Prevalence survey conducted in 2015 to understand how education, as a measure of socioeconomic status (SES), relates to obesity in Mongolia.*

*We found that women with lower education levels are more likely to be overweight or obese, while the opposite is true for men—those with higher education levels are more likely to be overweight or obese. These results reveal how obesity impacts men and women differently based on their social and economic circumstances. Our study underscores the need for targeted public health interventions, such as promoting affordable, nutritious diets and increasing access to physical activity programs, particularly for men in higher SES and women in lower SES groups. These insights provide valuable guidance for policymakers aiming to address the rising burden of obesity and its socioeconomic disparities in Mongolia and similar LMICs.*

**To the Reviewer 1**

We appreciate your invaluable comments and suggestions for the manuscript. We have revised the manuscript accordingly.

Major comments:

- **Comment 1:** One of the weaknesses of this study is the claim that the authors analyze socioeconomic inequality when they only use education as a ranking variable. Education is only a component of socioeconomic status (SES). I would recommend better constructing an SES index, which, among other factors, includes education.

***Response:*** *Thank you for your insightful comment regarding the use of education as the ranking variable. We agree with your comment that education represents only one dimension of SES and does not fully capture its nature, which also can be measured by income, occupation, wealth and household consumption. However, our decision to use education as a ranking variable was based on several factors: first, education is a commonly used and robust indicator of SES in health research, particularly in settings where reliable and accurate data on income or wealth are not available. Also, education is strongly associated with health outcomes and serves as a stable and easily measurable. Also, Bruno G et a. mentioned that if the central interest of the study is to measure the socioeconomic gradient in a particular health outcome, then the choice of indicator may not be crucial^1^.*

*Second, our dataset from Mongolian National Tuberculosis Prevalence survey constrained our ability to construct a more comprehensive SES index. Key variables necessary for a multidimensional SES index such as detailed income, occupation, and household wealth variables were either unavailable or incomplete in this dataset.*

*We agree with your suggestion that a more comprehensive SES index, incorporating multiple dimensions of socioeconomic status, would provide a more nuanced understanding of socioeconomic inequality. Future studies could address this limitation by collecting more detailed SES data to construct indices such as the Wealth Index or a composite SES score. Despite these limitations, we believe that our use of education as a ranking variable offers valuable insights into socioeconomic inequalities in overweight and obesity within the Mongolian context.*

*In order to clarify this limitation, we added the following sentences in the METHOD section and the limitation part of the DISCUSSION section in the manuscript.*

*Line 194-201, p-8,9*

*First, in Mongolia, one third of the population are herders whose livelihoods rely on farming and self-production, making it difficult to calculate the real income accurately [35]. Second, education is considered a reliable variable over time [36]. Education offers a more consistent and distinguishable indicator of socioeconomic status and access across different regions [30,34]. Numerous studies have stressed that education can be considered a measure of one’s socioeconomic status, and previous research found that the validation of self-reported education level was adequately complete and correct compared to self-reported income variables [37].*

*Line 411-413, p-19.*

*We used education as a main indicator variable which is a strong determinant of employment and income for adults [56].*

- **Comment 2:** From the title, the authors would focus (and maybe contribute) on decomposition approaches. Rather they do not even explain nor detail which decomposition method/approach they are using. The literature on decomposition methods in health is now very extensive. Each method has its assumptions, pros and cons. Thus, authors should include an entire subsection in the Methods section to provide further details about the decomposition approach followed. Furthermore, authors must argue why they are using such an approach versus other methods.

***Response:*** *Thank you for your critical and constructive feedback. We appreciate your observation regarding the need for greater clarity about the decomposition method used in our study. O’Donnell et al. explained this method well in the book and we provided that as a reference.*

*We have changed the title of the study as follows.*

*Education-related socioeconomic inequality in overweight and obesity among Mongolian adults*

*Also, we added a sentence as a subsection about the decomposition analysis in the METHOD part of the manuscript.*

*Line 241-243, p-10*

*We used regression-based decomposition for rank-dependent CI. The linear additive model is used for the analysis.*

**Comment 3:** The authors should make a strong case for studying the case of Mongolia. Given their current narrative, I cannot see what the study of Mongolia, as a case study, adds to the extensive literature on inequality and nutrition-related outcomes. It looks like the interest may reside in the fact that Mongolia transitioned to a market economy in the 1990s. However, the data used is cross-sectional from April 2014 to 2015. Thus, two recommendations: a) to use data before 1990 and compare before and after the market transition or b) to control for the year of birth in the analysis rather than age.

***Response:*** *Thank you for your thoughtful feedback and valuable recommendations. We appreciate your concern about the relevance of Mongolia as a case study in the context of inequality and nutrition-related outcomes and your suggestions for strengthening the narrative. However, as you noted, our study uses cross-sectional data collected in 2015, which limits our ability to directly assess pre- and post-transition dynamics. In response to your recommendation, we added the following paragraph in the introduction section. We revised the INTRODUCTION to articulate more clearly why Mongolia is a unique and important context for studying socioeconomic inequalities in overweight and obesity.*

*Line 111-125, p-5,6*

*Mongolia is a middle-income country, located in central Asia, with a population of 3.4 million in 2023, education level is high with over 95% of men and women attended primary school [10, 11]. Over the last three decades, Mongolia has undergone rapid economic growth, with its GDP per capita tripling from $4983 in 1990 to $12275 in 2023, due to foreign investment in the mining sector [12,13]. This economic development has been accompanied by rapid urbanization, with 70% of the population now residing in urban areas, and a shift from a traditional nomadic lifestyle to a more Westernized, sedentary way of living [14–16]. These societal changes have significantly influenced dietary patterns and physical activity levels. Previous studies have highlighted an excessive consumption of meat and refined grains, inadequate intake of fruits and vegetables, and dietary imbalances exacerbated by national food supply challenges. Additionally, physical activity level declined notably among both men and women [17,18]. For example, a study in Mongolia found that the prevalence of low physical activity increased from 8.2% in 2005 to 28.4% in 2019 [19]. Together, these shifts contribute to the rising prevalence of NCDs, including obesity [20].*

- **Comment 4:** Other variables: no discussion nor theory is guiding the inclusion of these variables. There must be at least some justification for the inclusion of each variable. As it is now, it seems more like a convenient exercise than a research-based analysis.

***Response:*** *Thank you for your valuable feedback regarding the need for a theoretical framework guiding the inclusion of variables in our analysis. We understand the importance of aligning variable selection with a robust theoretical concept. In this study, we utilized an already collected dataset from the Mongolian National Tuberculosis Prevalence Survey (MNTPS). As this dataset was not specifically designed for the study of socioeconomic inequalities in overweight and obesity, we were limited in our ability to incorporate theoretical considerations during the data collection phase.*

*However, we recognize the importance of justifying the inclusion of variables in our analysis. Some of our authors have published several studies regarding to social and educational inequality on health context. For your concern, we provided some publications of a similar method used for analysis.*

- *Dorjdagva J, Batbaatar E, Svensson M, Dorjsuren B, Batmunkh B, Kauhanen J. International Journal for Equity in Health. doi:10.1186/s12939-017-0572-4*
- *Dorjdagva J, Batbaatar E, Dorjsuren B, Kauhanen J. Explaining differences in education-related inequalities in health between urban and rural areas in Mongolia. Int J Equity Health. 2015;14(1):1-12. doi:10.1186/S12939-015-0281-9/FIGURES/3*
- *Dorjdagva J, Batbaatar E, Svensson M, Dorjsuren B, Kauhanen J. Catastrophic health expenditure and impoverishment in Mongolia. Int J Equity Health. 2016;15(1):1-9. doi:10.1186/S12939-016-0395-8/TABLES/4*
- **Comment 5:** The authors have done a very superficial literature review. I can identify at least three omitted papers belonging to this literature:

o Davillas, A., & Jones, A. M. (2020). Ex ante inequality of opportunity in health, decomposition and distributional analysis of biomarkers. Journal of Health Economics, 69, 102251. issn: 0167-6296..

o Nie, Peng; Ding, Lanlin; Jones, Andrew M. (2020) : Inequality of Opportunity in Bodyweight among Middle-Aged and Older Chinese: A Distributional Approach, IZA Discussion Papers, No. 13421, Institute of Labor Economics (IZA), Bonn

o Apostolos Davillas, Michaela Benzeval. Alternative measures to BMI: Exploring income-related inequalities in adiposity in Great Britain. Social Science & Medicine, Volume 166,2016. Pages 223-232, https://doi.org/10.1016/j.socscimed.2016.08.032.

o Joan Costa-Font, Joan Gil. What lies behind socio-economic inequalities in obesity in Spain? A decomposition approach. Food Policy. Volume 33, Issue 1,2008, Pages 61-73. https://doi.org/10.1016/j.foodpol.2007.05.005.

**Response:** *Thank you for your feedback regarding the literature review. Thank you for bringing this to our attention. We have included some of the articles you mentioned in the literature review.*

Other comments

• **Comment 6:** Many acronyms never defined, such as STEPS or NCDs

**Response 6:** *Thank you for pointing out some acronyms were not defined. We apologize for this oversight and appreciate your bringing it to our attention. We included list of abbreviation in the manuscript.*

*In the revised manuscript, we ensure that all acronyms are defined at their first mention.*

*Line 81-84, p-4*

*Although obesity is preventable and manageable, it is responsible for 5 million deaths globally from noncommunicable diseases (NCDs), such as cardiovascular disease and diabetes mellitus, and most of these deaths occur in LMICs [2].*

*In the previous manuscript, line 107-109*

*In Mongolia, the prevalence of overweight and obesity has been steadily increasing across all age groups and sexes. Four consecutive STEPS surveys revealed that the prevalence of overweight nearly doubled from 21.8% in 2005 to 49.4% in 2019 [12–14].*

*We corrected some numerical errors and rewritten this sentence as follows in the revised manuscript.*

*Line 127-130. P-6*

*Between 2005 and 2019 in Mongolia, the prevalence of overweight doubled, increasing from 18.2% to 48.0% among men and from 25.5% to 50.9% among women. During the same period, the overall prevalence of obesity rose significantly, from 10.2% to 18.5% [16,22,23]*

• **Comment 7:** Line 144-Page 6, clearly define what is the “inclusion criteria”

**Response 7:** *In the revised manuscript, we updated the text to define the inclusion criteria for participants in the study.*

*Line 168, p-7*

*The main inclusion criteria in the present study were that respondents be 18 years and or older.*

• **Comment 8:** Perform sensitivity of results when using another indicator for overweight and obesity, not just BMI

**Response 8**: *Thank you for your suggestion to perform a sensitivity analysis using an alternative indicator for overweight and obesity, such as waist circumference (WC) or waist-to-hip ratio (WHR). We agree that exploring additional measures beyond BMI would provide a more comprehensive assessment of obesity and its socioeconomic disparities. However, the dataset used for this study, the Mongolian National Tuberculosis Prevalence Survey (MNTPS), did not include data on alternative indicators such as WC or WHR. As a result, we are unable to perform the suggested sensitivity analysis. To address this, we included the following sentence in the manuscript.*

*Line 182-185, p-8*

*The previous study found that the WHO standard threshold of BMI cut-off point is appropriate for Mongolians, therefore, we used BMI cut-off points of 25-29.9 kg/m2 for overweight and BMI ≥ 30 kg/m2 for obesity [32].*

• **Comment 9:** Why are authors including Marital status in their analysis?

**Response 9:** *Thank you for your question regarding the inclusion of marital status in our analysis. Marital status is included based on its well-documented association with the risk of overweight and obesity.*

*We included in the DISCUSSION part of the revised manuscript to clarify why marital status is associated with inequality as follows.*

*Line 360-365, p-17*

*Marital status such as being single acted as a negative contributor to inequality, suggesting that single men may adopt healthier lifestyles or benefit from greater social stability. For example, a most recent systematic review revealed that married individuals have higher odds of developing obesity compared to unmarried individuals [49]. On the other hand, being single or re-entering marriage markets tends to lose weight due to behavior change and increased physical activities.*

• **Comment 10:** Is household income self-reported? If yes, how are authors dealing with the great risk of measurement error in this variable?

Response 10: *Thank you for raising this important concern regarding the potential measurement error in self-reported household income. As we utilized an existing dataset, we were unable to directly address or mitigate the risk of measurement error in the income variable. We acknowledge that self-reported income can be prone to inaccuracies due to recall bias, misreporting, or varying interpretations of income-related questions. Although we were limited by the constraints of the dataset, we appreciate your feedback, which has helped us transparently report this important methodological challenge.*

*We mentioned this limitation in the DISCUSSION section.*

*Line 409-411, p-19*

*However, this study has limitations. We used cross-sectional data from the tuberculosis prevalence survey, which did not allow us to perform a causal analysis and capture the relationship between individuals’ income and wealth differences.*

• **Comment 11:** In Figure 1, define the education levels, what is the third-level?

**Response 11:** *Thank you for pointing this out.* *In the revised manuscript, we have replaced the term third-level education with “tertiary education” for consistency and clarity. We hope this terminology update ensures greater clarity for our readers.*

• **Comment 12:** In Figure 2, the colours used for marital status and residual factors are very similar.

**Response 12:** *Thank you for your observation regarding the similarity of colors used for marital status and residual factors in Figure 2. The color has been updated in Figure 2.*

• **Comment 13:** In Figure 2, why is there no decomposition for women overweight?

**Response 13:** *Thank you for your observation regarding the absence of decomposition for women’s overweight in Figure 2. This omission is intentional, as being overweight among women was not significant in the concentration index analysis. Given the lack of significant socioeconomic inequality in this subgroup, further decomposition was not pursued to avoid overinterpreting non-significant results. We mentioned this decision in the RESULT section of the manuscript.*

*Line 281-282, p-13*

*The EI for overweight women was -0.004, which was not statistically significant, leading to its exclusion from further analysis.*

**Comments from Reviewer 2**

- **Comment 1:** This paper is well-executed and places itself effectively within the related literature. The methodology is sound, and the analysis is well conducted. However, the paper’s contribution to the field is not immediately clear, as it does not sufficiently emphasize how it advances current knowledge or offers new insights. To enhance its impact, the authors should focus on clearly articulating the study’s unique value and ensuring it is prominently highlighted in the discussion. Several areas could benefit from revision and clarification:

**Response 1:** *Thank you for your thoughtful and encouraging feedback on our study. We are pleased to hear that you find the paper well-executed and appreciate your constructive suggestions for improving its clarity and contribution to the field. We explicitly discussed how our analysis fills existing gaps in the literature, particularly in the context of socioeconomic inequalities in overweight and obesity in Mongolia, a setting with unique cultural, economic, and health dynamics. We emphasized how the study adds to the growing body of knowledge on the impacts of transitional economies on health outcomes, offering insights that may be relevant to similar contexts. Moreover, we discussed the policy implications of our findings, especially how they can inform targeted interventions to reduce health disparities in Mongolia and other countries experiencing rapid socioeconomic transitions. In order to ensure and clarify the statement and justification, we added and revised several parts in the INTRODUCTION and DISCUSSION parts of the manuscript.*

*A paragraph related to the country’s unique context and the impact of transitional economics on health was added in the INTRODUCTION section.*

*Line 111-125, p-5*

*Mongolia is a middle-income country, located in central Asia, with a population of 3.4 million in 2023. The education level is high with over 95% of men and women who attended primary school [10, 11]. Over the last three decades, Mongolia has undergone rapid economic growth, with its GDP per capita tripling from $4983 in 1990 to $12275 in 2023, due to foreign investment in the mining sector [12,13]. This economic development has been accompanied by rapid urbanization, with 70% of the population now residing in urban areas, and a shift from a traditional nomadic lifestyle to a more Westernized, sedentary way of living [14–16]. These societal changes have significantly influenced dietary patterns and physical activity levels. Previous studies have highlighted excessive consumption of meat and refined grains, inadequate intake of fruits and vegetables, and dietary imbalances exacerbated by national food supply challenges. Additionally, the physical activity level declined notably among both men and women [17,18]. For example, a study in Mongolia found that the prevalence of low physical activity increased from 8.2% in 2005 to 28.4% in 2019 [19]. Together, these shifts contribute to the rising prevalence of NCDs, including obesity [20].*

*The following sentences were added and rewritten in the DISCUSSION section.*

*Line 323-329, p-16*

*Our descriptive analysis revealed that the prevalence of obesity was higher among women (22.9%) than men (17.4%). Overweight was more common among individuals with higher education levels, with a significant education-related inequality observed in men but not in women. Although more women were obese overall, the concentration of obesity varied: men with higher education levels and women with lower education levels showed higher rates of obesity, but these gender difference did not hold for the obesity in overall population measure.*

*Line 330-331, p16*

*Socioeconomic, demographic, and behavioral contributing factors to inequalities with varying impacts based on gender.*

*Line 335-338, p-16*

*Studies in this context have also highlighted a positive association between higher SES and obesity among men, largely driven by increased access to calorie-dense food, reduced physical activities due to urbanization, and sedentary work patterns.*

*Line 340-345, p-19,20*

*Similarly, a study in Mongolia indicated an increased rate of physical inactivity in urban areas among men due to greater vehicle usage and a sedentary lifestyle, along with extremely high consumption of meat and sugar-sweetened beverages [16, 43]. Yuenan Su et al. found the prevalence of overweight, and obesity was higher among high SES participants of the research conducted in Inner Mongolia, due to an increased unhealthy diet with intake of red meat and processed food [42].*

*Line 360-368, p-17,18*

*Marital status such as being single acted as a negative contributor to inequality, suggesting that single men may adopt healthier lifestyles or benefit from greater social stability. For example, a most recent systematic review revealed that married individuals have higher odds of developing obesity compared to unmarried individuals [49]. On the other hand, being single in or re-entering marriage markets tends to lose weight due to behavior change and increased physical activities.*

*Conversely, among women, the prevalence of obesity is higher in lower socioeconomic groups. This pattern is frequently observed in HICs, and in some LMICs where socioeconomic disadvantage disproportionately impacts women's health.*

*Line 375-379, p-18*

*For example, studies conducted in Mongolia revealed that the women in rural areas consume markedly less fruits and vegetables during both winter and summer, and consuming red meat and refined grains was extremely high compared to urban counterparts [25]. Additionally, challenges in healthcare access and education-related inequalities in healthcare utilization were identified [55].*

*Line 387-387, p-18*

*Domestic and caregiving duties can result in additional time constraints for women in rural areas, which limits their opportunities for physical activity [45].*

- **Comment 2: Rank Variable for SES:** While using years of schooling to measure socioeconomic status can provide valuable insights, it is typically a fallback when income data is unavailable. Income is generally a more robust and sensitive rank variable due to its greater variability across populations and its consistent relationship with obesity. The statement on p.7, line 164, referring to education as “stable over time,” needs further justification. Stability is not always an advantage in this context, as income's dynamic nature reflects current socioeconomic conditions more accurately. Results using income as the rank variable should be presented and compared, and the preference for education over income should be better justified.

**Response 2:** *Thank you for your insightful comments regarding the use of years of schooling as the rank variable for socioeconomic status (SES). We agree with your comment that education represents only one dimension of SES and does not fully capture its nature, which also can be measured by income. While income is often preferred as an SES measure due to its dynamic nature and direct association with current socioeconomic conditions, its use in the Mongolian context is limited by measurement challenges. Self-reported income is prone to recall bias and variability, especially in populations like herders and self-producers, whose livelihoods do not follow standard economic patterns. Education, in contrast, provides a stable and robust indicator of long-term socioeconomic position, reflecting access to resources and opportunities across one’s life course.*

*We have included a detailed justification in the METHODS section of the manuscript.*

*Line 190-201, p-8,9*

*The concentration index, a rank-dependent method for measuring socioeconomic-related health inequalities. Education was used as a measure of SES in relation to being overweight and obese, as it is considered a stable variable over time [36]. In Mongolia, one third of the population are herders whose livelihoods rely on farming and self-production, making it difficult to calculate real income accurately [35]. Therefore, we selected education as the measure of socioeconomic inequality in overweight and obesity. Education offers a more consistent and distinguishable indicator of socioeconomic status and access across different regions [30,34]. Numerous studies have stressed that education can be considered a measure of one’s socioeconomic status, and previous research found that the validation of self-reported education level was adequately complete and correct compared to self-reported income variables [37].*

- **Comment 3:** Definition of "Location": The term "location" on p.7, line 170, seems to imply detailed geographic information, but the study only differentiates between rural and urban areas. This distinction should be clarified to avoid misinterpretation.

**Response 3:** *Thank you for highlighting the need to clarify the definition of "location". In our study, "location" refers specifically to the distinction between rural and urban areas. We opted to use the term "location" to align with the terminology used in previous research, ensuring consistency and comparability across studies. This approach facilitates the integration of our findings into the broader body of literature on socioeconomic inequalities in health.*

- **Comment 4:** Alcohol Consumption Variable: The variable indicating alcohol consumption (p.7, line 177) appears to consider whether a person consumed alcohol more than once in the past year. This threshold is unlikely to meaningfully impact SES inequality in obesity. Alternative measures, such as binge drinking or more frequent recent alcohol use, would be more appropriate.

**Response 4:** *Thank you for your insightful comment regarding the alcohol consumption variable. Unfortunately, due to the limitations of the data used in this study, we were constrained to the available measure of alcohol consumption, which captures whether a person consumed alcohol more than once in the past year. While this measure may provide a general indicator of alcohol use, we acknowledge that it is insufficient to fully explore the nuanced relationship between alcohol consumption patterns and obesity. However, data from the Centers for Disease Control and Prevention (CDC) indicate that in 2018, approximately 33.7% of U.S. adults aged 18 and over did not consume alcohol in the past year. This supports the categorization of alcohol consumption patterns in our study.*

- **Comment 5:** Decomposition Analysis: The decomposition results indicate contributions greater than 100%. This needs to be explained thoroughly, including how these results are calculated. In general, tables should include all key components (e.g., concentration index, elasticities, and factor contributions) for transparency and completeness.
- **Response 5:** *Thank you for your comment regarding the decomposition analysis results and the observed contributions exceeding 100%. We appreciate the opportunity to clarify this aspect of the analysis and to enhance transparency in presenting the results. Some factors contribute positively to inequality while others contribute negatively. A negative contribution from one factor can amplify the relative share of other factors, potentially pushing their contributions above 100%.*

*We added the following sentences in the RESULT section of the manuscript.*

*Line 299-301, p-14*

*It is important to note that the contributions of some determinants to the CI may exceed 100%. However, the sum of contributions of all the determinants equals 100%.*

- **Comment 6:** The sentence on p.4, lines 87–89, is unclear and needs to be rewritten for better comprehension.

**Response 6:** *Thank you for pointing out the need for clarity in the sentence on p.4, lines 87–89. We revised the sentence to ensure it is clear and comprehensible.*

*The original sentence reads:*

*Obesity is a complex condition influenced by individual predispositions, behavioral factors (such as diet, physical activity, smoking, and alcohol consumption), and social determinants such as cultural and socioenvironmental factors [3]. These factors are distributed unevenly across socioeconomic status (SES), leading to an inequitable burden of overweight and obesity that disproportionately affects certain population groups [4-6].*

*We have rewritten it as follows:*

*Line 87-96, p4*

*Obesity is caused by a complex interplay of factors, including genetic, psychological, metabolic, behavioral and social1. These factors are distributed unevenly across socioeconomic status (SES), leading to an inequitable burden of overweight and obesity that disproportionately affects certain population groups [3]. Differences between people in SES, lifestyle, social inequality, cultural practices, and living environment have significant roles in weight gain [4]. Socioeconomic status (SES) is a broad concept primarily determined by income, level of education, and occupational status. It is closely linked to perceptions of social standing and access to resources and opportunities that contribute to overall well-being. [3]. The association between SES and obesity across genders can differ between and within countries, depending on the country’s level of socioeconomic development [5].*

- **Comment 7:** Paper Structure: Include a paragraph at the end of the introduction section outlining the structure of the paper, guiding readers through its organization.

**Response 7:** *Thank you for your suggestion to include a paragraph at the end of the introduction outlining the structure of the paper. We appreciate the value of providing readers with a roadmap to navigate the manuscript. However, we followed the formatting and content requirements of the journal, which do not explicitly request or recommend such section.*

- **Comment 8:** Discussion Section: A significant portion of the discussion section would be better suited for the introduction and literature review, where it could help frame the study’s research question and relevance.

**Response 8:** *Thank you for your feedback regarding the organization of the discussion section. In the revised manuscript, we have addressed this concern by moving the content related to the country context from the discussion section of the previous manuscript to the introduction section. This change ensures that the introduction provides a comprehensive background and frames the study’s research question and relevance more effectively.*

*The revised discussion section now focuses more specifically on interpreting the findings, comparing them with existing literature, highlighting the study’s contributions, discussing policy implications, and addressing limitations. This restructuring aligns with the journal's expectations and enhances the clarity and focus of the manuscript.*

*The original sentence reads in the DISCUSSION section:*

*Line 287-292, p-15*

*Overweight and obesity result from an energy imbalance between calories consumed and calories expended [10]. Recent shifts in dietary patterns and physical activity in LMICs, including Mongolia, highlight the impact of economic transitions on health. Since the transition from a centrally planned economy to a market economy in the 1990s, Mongolia has experienced rapid changes in nutrition and lifestyle, leading to increased consumption of high-calorie, low-nutrient foods and a more sedentary lifestyle [28, 16].*

*We have rewritten it as follows in the INTRODUCTION section:*

*Line 111-125, p-5*

*Mongolia is a middle-income country, located in central Asia, with a population of 3.4 million in 2023. The education level is high with over 95% of men and women who attended primary school [10, 11]. Over the last three decades, Mongolia has undergone rapid economic growth, with its GDP per capita tripling from $4983 in 1990 to $12275 in 2023, due to foreign investment in the mining sector [12,13]. This economic development has been accompanied by rapid urbanization, with 70% of the population now residing in urban areas, and a shift from a traditional nomadic lifestyle to a more Westernized, sedentary way of living [14–16]. These societal changes have significantly influenced dietary patterns and physical activity levels. Previous studies have highlighted excessive consumption of meat and refined grains, inadequate intake of fruits and vegetables, and dietary imbalances exacerbated by national food supply challenges. Additionally, physical activity level declined notably among both men and women [17,18]. For example, a study in Mongolia found that the prevalence of low physical activity increased from 8.2% in 2005 to 28.4% in 2019 [19]. Together, these shifts contribute to the rising prevalence of NCDs, including obesity [20].*

- **Comment 9:** Conclusion: The conclusion is too brief and does not adequately summarize the study’s findings. It should discuss the key insights, such as why obesity is more prevalent among women in lower SES groups while the reverse is true for men, and explicitly state what questions the study addresses.

**Response 9:** *Thank you for your feedback regarding the conclusion. However, we followed the formatting and content requirements of the scientific writing style, which do not explicitly request or recommend detailed conclusions.*

- **Comment 10:** Policy Recommendations: The policy recommendations are underdeveloped and should provide more detailed, actionable suggestions based on the study’s findings.

**Response 10:** *Thank you for your feedback. We agree that this section could be expanded to provide more detailed and actionable suggestions directly informed by the study’s findings.*

*The original sentence reads in the DISCUSSION section:*

*Line 336-340, p-17*

*Our findings indicate that obesity-related policies should focus on high-risk groups, including men with higher education and income, women with lower education, rural residents, and older individuals. Policy interventions should aim to address environmental factors such as nutrition security, food reformulation, active transportation infrastructure, and marketing regulations.*

*We rewrote the policy recommendations as follows in the DISCUSSION section:*

*Line 395-407, p-19*

*Our findings indicate the need for gender-sensitive, evidence-based obesity-related policies to address the socioeconomic inequalities driving overweight and obesity in Mongolia should focus on high-risk groups, including men with higher education and income, women with lower education, rural residents, and older individuals. Implement programs to reduce sedentary lifestyles, promote healthier diets, and increase physical activity through worksite wellness initiatives, education campaigns, and active transport infrastructure for economically advantaged men, and socioeconomically disadvantaged women, policies should focus on improving health literacy, providing affordable nutritious food options, and developing community-based physical activity programs. Overall, at the country level, regulation of food marketing, encouraging reformulation of processed foods, and promoting sustainable urban infrastructure that supports physical activities (walkable cities and cycling roads etc.,) are crucial. Also, the development of targeted programs focusing on older women and addressing poverty, pension and healthcare access to improve diet and lifestyle is important.*
