# Supplementary material for: Education-related inequality in overweight and obesity among Mongolian adults
Source: PLOS Glob Public Health. 2025 Jun 17;5(6):e0004723. doi: 10.1371/journal.pgph.0004723 (PMC12173372; doi:10.1371/journal.pgph.0004723)
Supplement: Attachment — Submitted filename: Response letter to reviewers_R2.docx [file pgph.0004723.s004.docx]

PGPH-D-24-02309R1

Dear Reviewers,

Thank you for the opportunity to submit a revised version of our manuscript, *“Education-related socioeconomic inequality in overweight and obesity among Mongolian adults”* for consideration in *PLOS Global Public Health*.

We sincerely appreciate the time and effort that you and the reviewers have dedicated to providing valuable feedback on our work. We are grateful for the reviewers’ insightful comments, which have helped us strengthen the manuscript. In response, we have carefully revised the manuscript to incorporate most of the suggested changes. All modifications have been tracked within the document for ease of reference.

Sincerely,

Authors

**To the Reviewer**

We appreciate your invaluable comments and suggestions for the manuscript. We have revised the manuscript accordingly. The authors have agreed to have clearer statements in the Introduction and Discussion sections of the manuscript after your comments to add clarity of the manuscript. Therefore, we made minor changes and additions to the Introduction and Discussion parts. We believe that the manuscript is now more logical and has more added value after these changes. We are truly grateful for your patience and professional comments to improve the manuscript.

**Comment 1:**

Regarding your response to comment 4. I must say, it is tendentious to cite three articles by the same authors as examples of publications where a similar method was used for the analysis. Moreover, the authors heavily justify the lack of discussion about the variables included in their analysis based on the dataset used. There are now several methods based on machine learning designed precisely to avoid cherry-picking. Thus, I strongly recommend to, at least, include a brief justification for the inclusion of the variables mentioned in the “other variables” subsection.

***Response 1:***

*We appreciate the reviewer’s concern regarding the justification of a variable selection and the methodological approach used in our study. We totally agree with you on the availability of the newly emerging methods to analyze health inequality. The authors agreed to apply the method you suggested in further studies. Based on the data availability in this particular study and the nature of the health economics field, however, the Concentration index is recognized as the most convenient method to evaluate inequality in health and health care utilization. For this reason, we have applied the Erreyger’s concentration index using the variables “education” and “health outcome (overweight and obesity)”. To identify the contributing factors to the concentration index, we employed the decomposition analysis using age, sex, household income, marital status, employment status, location (urban, rural areas), smoking status and alcohol consumption variables which we have categorized them as “other variable” in the manuscript. However, we completely agree that there might be some ambiguity in the variable description part which has poor justifications of usage of the variables in this study. We have elaborated variable descriptions and some justifications regarding your comments in the Materials and methods section of the manuscript. Also, the “other variables” has been named “independent variables” in the Materials and methods part for the sake of clarity. Also, we added following sentence in this subsection.*

*Line 172-175, p7,8*

*Independent variables*

*In this study, in addition to the concentration analysis, the decomposition analysis was conducted to identify potential demographic and socio-economic contributing factors to the concentration index. These contributing factors are regarded as independent variables here.*

**Comment 2:** It is somewhat surprising that age is the main driver of education-related inequality in obesity among women. Please conduct a sensitivity analysis excluding age to test whether nutrition-related inequality is driven by education.

***Response 2:***

*We are truly thankful for your constructive comment on the manuscript. We completely agree with your valuable comment, and it helps us find an inexplicit statement in the manuscript. The decomposition analysis we employed in this study enables us to extract the contributions of each demographic and socio-economic factor to education-related inequality in obesity and overweight. The results show that age is one of the main contributors to the total inequality in obesity.*

*It is important to mention that the size of contributions of socio-economic factors to the concentration index can be easily obtained by subtracting age’s contribution from the total contribution of all factors to the concentration index.*

*We just found out that the results of the decomposition analysis weren’t explained in an appropriate way in the Results part of the manuscript which might cause unambiguity. In the method section we have cited the source of the well-known method of decomposition analysis. We have rewritten the results of decomposition analysis in the Results part of the manuscript according to your comment.*

*The following sentences were rewritten in the RESULT section.*

*The original sentence reads:*

*Line 298-307, p14*

*However, the sum of contributions of all the determinants equals 100%. For women, the significant positive contributors to education-related inequality in obesity were age (109.84%), education level (15.18%), and rural residence (8.0%). Negative contributors were income (-17.03) and alcohol consumption (-10.28%) For men, significant positive contributors to inequality in overweight individuals included education (98.37%), employment (15.95%), income (24.08%), alcohol consumption (6.2%), and smoking (5.6%). For obesity, significant positive contributors included income (21.2%), education (100.4%), employment (9.3%) and smoking (4.72%), whereas age (-33.88%) and marital status (-1.6%) made significant negative contributions to the total inequality.*

*We have rewritten it as follows:*

*Line 264-267, p12*

*Conversely, income was concentrated among higher-educated women (CI = 0.012),*

*which contributes 17% to education-related inequality in obesity among women If income was equally distributed across education groups among women, estimated degree of education-related inequality in obesity would be 17% higher. The results also show that a positive association between income and obesity among women.*

*Line 271-274, p13*

*Education-related inequalities in overweight and obesity among men in Mongolia were mainly caused by education, employment status, and income, except the contribution of age.*

*While obesity was concentrated among lower-educated women which was caused by education, income, alcohol use, and location, except the contribution of age.*
